# Gender‐inclusive language in midwifery and perinatal services: A guide and argument for justice

**DOI:** 10.1111/birt.12844

**Published:** 2024-06-01

**Authors:** Sally Pezaro, John Pendleton, Rodante van der Waal, Sarah LaChance Adams, Mario J. D. S. Santos, Ash Bainbridge, Krishna Istha, Zan Maeder, John Gilmore, Jeannine Webster, Bunty Lai‐Boyd, Anne Marie Brennan, Elizabeth Newnham

**Affiliations:** ^1^ Research Centre for Healthcare and Communities Coventry University Coventry UK; ^2^ The University of Notre Dame Fremantle Western Australia Australia; ^3^ Faculty of Health, Education, & Society University of Northampton Northampton UK; ^4^ Care Ethics Department University for Humanistic Studies Utrecht The Netherlands; ^5^ Independent Midwife Bristol UK; ^6^ The Florida Blue Center for Ethics University of North Florida Jacksonville Florida USA; ^7^ Department of Sociology Universidade da Beira Interior Covilhã Portugal; ^8^ Iscte ‐ Instituto Universitário de Lisboa, CIES‐IUL Lisbon Portugal; ^9^ Three Counties School of Nursing and Midwifery University of Worcester Worcester UK; ^10^ https://www.krishnaistha.com; ^11^ Queer Doula Adelaide Australia; ^12^ School of Nursing Midwifery and Health Systems University College Dublin Dublin Ireland; ^13^ Dublin Ireland; ^14^ School of Nursing and Midwifery University of Newcastle Newcastle New South Wales Australia

**Keywords:** equity, gender, inclusive language, intersectional feminism, midwifery, pregnancy, reproductive justice

## Abstract

Effective communication in relation to pregnancy and birth is crucial to quality care. A recent focus in reproductive healthcare on “sexed language” reflects an ideology of unchangeable sex binary and fear of erasure, from both cisgender women and the profession of midwifery. In this paper, we highlight how privileging sexed language causes harm to all who birth—including pregnant trans, gender diverse, and non‐binary people—and is, therefore, unethical and incompatible with the principles of midwifery. We show how this argument, which conflates midwifery with essentialist thinking, is unstable, and perpetuates and misappropriates midwifery's marginalized status. We also explore how sex and gender essentialism can be understood as colonialist, heteropatriarchal, and universalist, and therefore, reinforcing of these harmful principles. Midwifery has both the opportunity and duty to uphold reproductive justice. Midwifery can be a leader in the decolonization of childbirth and in defending the rights of all childbearing people, the majority of whom are cisgender women. As the systemwide use of inclusive language is central to this commitment, we offer guidance in relation to how inclusive language in perinatal and midwifery services may be realized.

## Graphical abstract

A recent focus in reproductive healthcare on “sexed language” reflects an ideology of unchangeable sex‐binary and fear of erasure, from both cisgender women and the profession of midwifery. In this paper, we highlight how privileging sexed language causes harm to all who birth—including pregnant cisgender women, trans, gender diverse, and non‐binary people—and is therefore unethical and incompatible with the principles of midwifery.

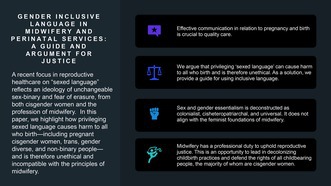

## 1 INTRODUCTION

The notion of childbearing having a necessary or logical belonging within the nuclear two‐parent family initiated by heterosexual couples whose gender has a normative relationship with their sex assigned at birth is a recent development in our human history, and one still inconsistently observed around the globe.^1^ Indeed, community and extended family are often as, if not more important. Yet, more recently and particularly in the Global North, perinatal and midwifery services have been positioned as “woman” centered and understood exclusively in heteropatriarchal and cisnormative terms.^2^ There is increasing recognition that such understandings are colonial in nature and do not acknowledge gender diversity.^3^, ^4^, ^5^ Thus there have been calls for reform through the development of inclusive guidelines and policies to be reflective of all who birth.^2^ However, there is a conservative counter‐resistance calling for the continued use of “sexed language”.^6^ In this paper, we will illustrate how such a move inadvertently reinforces the structures that presently oppress all birthing people, the majority of whom are cisgender women, and midwifery itself.

Midwifery with its unique body of knowledge, skills, and professional attitudes drawn from disciplines such as science and sociology has the potential to avert the majority of perinatal mortalities globally, and is practiced by midwives within a professional framework of autonomy, partnership, ethics, and accountability.^7^ Yet, the use of ‘sexed language’ undermines midwifery's particular commitment to reproductive justice^8^ and feminist ethics.^9^, ^10^, ^11^, ^12^ We highlight that midwifery instead has an opportunity to be effective in its advocacy of human and reproductive rights for all by embracing inclusive language to reflect its intersectional commitment to reproductive justice.^8^ Ultimately, midwifery has an ethical duty and opportunity to lead in gender decolonization and reproductive justice through the use of inclusive language. We conclude with guidance as to how this may be realized.

## 2 DEFINITIONS

In this article, the word ‘trans’ is used as an umbrella term to describe people whose experience of their gender differs from the sex recorded on their birth certificate, based on the appearance of external genitalia.^13^ ‘Cis’—short for ‘cisgender’—is used to describe people whose gender identity corresponds with the sex registered for them at birth. As language evolves, these definitions may quickly date and gender can be fluid. Therefore, we will predominantly use the alternate umbrella term “gender diverse” to describe non‐cisgender people, some of whom will be non‐binary in that they do not identify as either a man or a woman exclusively. One's sex may be defined as a spectrum of traits relating to one's hormones, chromosomes, reproductive anatomy, genitalia, and hormonal/gene expression,^14^, ^15^, ^16^, ^17^ though it is often referred to as being male or female in binary terms. We also acknowledge intersex individuals who may have genitalia outside the male–female binary and identify as trans or not.^18^ Gender is socially constructed and defined by the individual as to how they perceive themselves (e.g., woman/man/non‐binary), which in turn determines their behavior, performance, expression, and how society perceives them.^19^

## 3 POSITIONALITY

We are a coalition of midwives, midwifery students, trans, queer, gender diverse, and cisgender professionals. We are also academics in midwifery, sociology, and philosophy. Some have experienced the anti‐trans rhetoric apparent in the midwifery profession,^2^ which has influenced the way we practice and articulate our position in that we are motivated to seek reproductive justice where we see injustice. Whilst we have attempted to include a diversity of voices, we recognize that we largely offer a Global North perspective and thus those emerging from Indigenous voices and settler‐colonial societies in the Global South may not be adequately reflected.

## 4 SEXED LANGUAGE AND BODILY HARM

A recent paper co‐authored with midwives argues the importance of privileging sexed language for midwifery and perinatal care, suggesting its necessity for “effective communication”, and the avoidance of “confusion” when it comes to the delivery of care.^6^ There is an urgent need to understand the ethical implications when such exclusion of gender diversity is mobilized and imposed onto midwifery as a profession. If midwifery is committed to the principle of reproductive justice, then it has an ethical imperative to use gender‐inclusive language, because the use of exclusive language in perinatal care ultimately reproduces the notion that gender is determined by biological sex and restricts ideas about who can experience pregnancy and birth. Ultimately, harmful stereotypes and biases are propagated by such exclusive language,^20^ and as the paradigm case of Sam, a 32‐year‐old man demonstrates, a myopic focus on sexed language and the narrow classifications which follow can cause significant harm and death.

Sam presented to the hospital with abdominal pain and a positive home pregnancy test. He shared that he was a trans man. Sam's labor was not recognized by staff because of systems, biases, and stereotypes related to his gender presentation and identity. On evaluation by an emergency physician several hours later, a cord prolapse was detected. Sam's baby died. If Sam's care had instead been driven by pregnancy algorithms noting the organs he had in situ, the prolapsed cord may have been detected in time to prevent fetal death.^21^ Instead, the reinscribing of the gender/sex binary in this case and no doubt others reinforced by the use of exclusive language at a systemwide level caused harm. Such failure to recognize the complexities and intersectionality of people as individuals not only contravenes the principle of non‐maleficence,^22^ but arguably situates perinatal staff as agents of harm rather than agents of care. Though more robust research is required, the harm of such exclusionary practices is measurable.^3^, ^23^ The resultant avoidance of care‐seeking is linked to direct harm, which can be perpetuated by microaggressions, discrimination, and minority stress exposure in healthcare services.^2^, ^24^, ^25^, ^26^ Evidently, where perinatal services are rigidly gendered, worse health outcomes occur.^27^, ^28^, ^29^, ^30^

Groups opposing the use of inclusive language in perinatal services generally purport unforeseen deleterious consequences,^6^ noting ‘sexed language’ as the primary determination of access to healthcare, directly related to one's sex assigned at birth which is binary, immutable, and unchangeable.^31^ Yet, where policies in relation to language are exclusionary, language is used in fact to discriminate, abuse, police, marginalize, disrupt, and destabilize individuals and communities, and incorrectly infer pathology.^32^ “Sexed” language disregards the reality that bodies change continuously, and considerable variation exists within the categories of both sex and gender, neither of which are binary.^14^, ^15^, ^16^, ^17^, ^19^ Moreover, the constitution of the “sexed” body is detrimentally bound up with the medicalization of women's bodies and the eradication of gender‐diverse people's identities.^33^, ^34^ When we understand biological sex and gender as neither binary nor immutable, we reveal the need to capture diversity (e.g., change in any of the main components of biological sex) more accurately through inclusive approaches in all areas. These include research leading to more empirical, accurate, and just knowledge,^35^ individualized care, clinical outcomes, medical records, and human rights.

While epidemiological data remains largely uncaptured and likely underestimated, the number of people in the Global North identifying as gender diverse is increasing,^36^ much like the numbers of left‐handed people did when society became more inclusive of such populations.^37^ Thus, more gender‐diverse people will need high‐quality perinatal care, particularly as the harmful practice of sterilization, where the idea of pregnancy in these populations was unthinkable—is no longer encouraged.^38^ With such evolving populations and recognition of the diversity in childbearing people, inclusive approaches are urgently required for the avoidance of future harm. Indeed, we will argue that an inclusive perinatal service will not only benefit gender diverse folk, but all who birth, the majority of whom are cisgender women.

## 5 MIDWIFERY, ESSENTIALISM, AND FEMINISM

Whilst this paper is only able to provide a narrow discussion of feminism and midwifery, it has been described as an intrinsically feminist profession, “a manifestation of feminism in action”,^39^ and many midwives see themselves as feminists working within, and resisting the systems shaped by medical, industrial, patriarchal, colonizing, and neoliberal influences. Midwifery has been tasked as a feminist resistance to medicalization,^40^, ^41^ with its philosophies of practice such as ‘woman‐centred’ care and a focus on the ‘mother‐midwife’ relationship.^42^ Yet, while some feminists were exploring how the maternal instinct may be used to galvanize societal change,^43^ second‐wave feminists fought for reproductive and economic freedoms.^44^ Although pregnancy and childbirth were given some attention in the social sciences,^41^, ^45^, ^46^ intense ambivalence toward pregnancy, childbirth, and mothering is demonstrated in feminist theoretical discourse. Some philosophers joined the race to free women from the “tyranny of reproduction”^47^; the obligations of motherhood and marriage and, later, from biology itself, even while acknowledging that sexual difference can be affirming.^48^ The French philosophers of “sexual difference” such as Hélène Cixous, Luce Irigaray, and Julia Kristeva affirmed existential differences between the female body and women's' lives in comparison to male bodies and the lives of men. Australian philosopher Elizabeth Grosz also acknowledged the reality of bodily difference between the sexes, but recognized how these are not immutable nor biologically pre‐ordained.^49^ Indeed, Grosz documented a vitalism in being (in life) that propels the body to always become other, change, and generate the new, articulating the body as sociocultural, inscribed, and marked by power in a way that transcends binary logic.^49^ Sexual difference has been often misunderstood and misused as a rigid essentialist dichotomy between sexes, rather than as an affirmation of difference between sex and gender to expand on differences into further fluidity and plurality. In the United States of America, the essentialist and feminist theologist Mary Daly, now recognized as a main theorist of trans‐exclusive radical feminism, wrote the critique *Gyn/Ecology* on the medicalization of pregnancy and childbirth as a conscious attempt of medicine to take power over female bodies, actively suppressing their innate female power.^50^ However, now, many intersectional and postcolonial feminists discount the usefulness of generalizing terms (e.g., ‘sisterhood’ or ‘womanhood’) to capture experiences like childbirth and patriarchal suppression, since these terms not only deny the existence of gender‐diverse experiences, but also erase important differences between people identifying as women, such as race and class, and hence perpetuate ongoing marginalization through assumptions rooted in essentialism.^51^, ^52^ So too must midwifery and perinatal services move past such essentialist assumptions if they are to pursue equality for all who access their care.

Essentialist philosophies in midwifery have further been circumscribed through laying bare the history of perinatal care. For example, the “man midwives” of the late 17th century onwards,^53^, ^54^ forerunners to the modern‐day obstetrician, commodified childbirth as a lucrative revenue stream and used it to assert their technical and objective superiority with the invention of instruments such as forceps. This is contrasted with the undisturbed physiological birth supported by midwives. Their legacy of embodied knowledge means that midwives' work was also invested with the wisdom of women who had gone before and supported them, coupled with an altruistic responsibility to carry the profession forward and safeguard it for future generations. Such motivation continues to be cited as a reason for student midwives seeking to join the profession today.^55^, ^56^, ^57^ For many, midwifery exemplifies this resistance to the social marginalization of women‐led professions against patriarchy. However, if we describe patriarchy as a systemic and complex network of relations, within which cis men are the most privileged overall,^58^ we understand the enforcement of a naturalized gender binary as most harmful to all who are not cis men—that is, patriarchy is most harmful to both women and gender‐diverse people. Despite this, the struggle for midwifery's integrity is sometimes misunderstood as a fight “for women by women”—a position which is simplistic in its failure to acknowledge either the gender/sex spectrum or the intersectional argument that “women/woman” is not a discrete category.

For a time, though harmful, essentialism^59^, ^60^ was used as a strategy for promoting professional autonomy in midwifery. A late twentieth‐century counterculture in countries such as the United Kingdom (UK), based on a notion of “shared womanhood”, challenged obstetric hegemony, leading to a recentering of midwives as autonomous practitioners able to facilitate choice and control for women^61^ and, for a period of time managed to forestall encroaching obstetric interventions. It is, thus, not surprising that securing “sexed language” is currently seen by some as intrinsic to the continued success of resistance against obstetrics and medicalization. In this way, contemporary midwifery in the Global North attempts to secure an exclusive professional space. For instance, an ethnography of home births illustrates how not only midwives but also doulas make use of an essentialist discourse to resist the patriarchal appropriation of labor and birth by obstetrics and by the system of “maternity” care more broadly.^40^ In such narratives, women are encouraged to claim their authority over birth, reinstating the feminine back into the birth setting. Nevertheless, conflating midwifery with such essentialist thinking through the use of ‘sexed language’ will only perpetuate and misappropriate midwives' marginalized status, because this language further reinforces the very essentialism we will subsequently demonstrate causes harm.^62^

Midwifery as a political project also finds itself trying to navigate a nexus of power that must evolve, while reconciling the integrity of its unique system of beliefs, and discrete professional identities. Within a world structured *by* and *for* the benefit of patriarchy, it has been understandable and justifiable for midwives to have been resistant toward encroachments upon their professional expertise, particularly given that they have been identified as key to reductions in perinatal mortalities worldwide.^63^, ^64^ It is argued that midwifery has an overly denigrated professional identity,^65^ making it more vulnerable in being forced to defend a profession already under attack.^66^ In the face of changes to the provision of perinatal care (e.g., acknowledgment of gender diversity and inclusion in childbearing), it is perhaps not surprising that some seek to assert that inclusive language erases cisgender women.^2^ Just like midwifery has always been “erased” (i.e., marginalized), inclusive language dilutes the ability of perinatal services to label and make visible the oppression of women. This argument relies, however, on the notion that the unproblematized feminization of pregnancy is good for cisgender women, while complying with the patriarchal imperative that women shoulder all reproductive labor. Ultimately, the feminization (or indeed masculinization) of any single act, item, or behavior serves only to reinforce the cisheteronormativity which upholds patriarchy and is thus not only homophobic and transphobic but also misogynistic.

Evidently, providing “care for women by women” as a form of “resistance”^41^ has been an insufficient response to the ongoing encroachment of obstetric surveillance and intervention thus far.^59^ All birthing people continue to experience gender‐related oppression through patriarchy.^67^ The obstetric dominance of childbearing is accelerating once again, with a doubling of cesarean rates in many nations to over 50% of all births.^68^ There is also growing international concern over increased perinatal mortality and morbidity from unnecessary operative births.^69^ These escalating rates of obstetric interventions worldwide continue in spite of foregrounding essentialism and cisheteronormativity in midwifery.^2^ Clearly the use of sexed terms also does not provide a safe space from patriarchal oppression. Thus, approaches which instead dismantle sexist logic, rather than rely upon them, are now required.

The sexed language argument understands women to be facing the threat of erasure, not by the patriarchy, but by gender‐diverse people. It employs a scarcity narrative,^70^ wherein the safety of cisgender women cannot be shared with gender diverse people, rather than recognizing that both groups are oppressed by the same patriarchal and colonial influences that remain starkly present in the inequity of perinatal services and outcomes.^2^, ^71^ These influences play out in such instances where, although the use of both the words ‘man/men’ and ‘woman/women’ are problematic in inclusive healthcare guidance for example, the word ‘man/men’ is less contested and more resilient to removal from policy. Gender non‐conforming individuals are substituted for the true threat—the patriarchal structures that oppress multiple marginalized groups. This oppression becomes clear as we demonstrate the role of colonialism in the next section. We also foreground decolonial, intersectional feminism, and reproductive justice, which are key to the implementation of professional policy and practice in meeting the ethical imperative for gender‐inclusive language in perinatal care.

## 6 MIDWIFERY, ESSENTIALISM, AND COLONIALISM

An unnatural sex/gender binary has been aggressively inscribed by colonizing power structures and systems to position white heteropatriarchy as the pinnacle of civilization.^72^, ^73^ This binary is also racialized and sexualized at its foundations.^74^, ^75^ Outside the white, colonial Global North, histories, and understandings of gender in relation to birthing and parenthood are complex and rich. For example, in Hawaii, many māhū people embrace the feminine and masculine traits embodied in everyone. Before colonization, many more were respected as caretakers, healers, and teachers.^76^ In Australia, Sistergirls (Indigenous transgender people with a female spirit whose bodies were considered male at birth) look after children and family.^76^ In India, more hijras (the ‘third gender’) are reclaiming ‘mother’ to make this gendered term trans, third gender, and non‐binary inclusive, aspiring to a future where they are identified not as ‘a hijra mother’, but as ‘a mother’.^77^ In British Columbia, marginalization and erasure of Two‐Spirit people and their roles in Aboriginal culture have resulted in discriminatory pregnancy experiences,^78^ and a report on anti‐Indigenous racism in British Columbia's healthcare system entirely omitted the words ‘Two Spirit’, therefore denying the existence of more than two genders.^79^ In Aotearoa New Zealand, the pre‐colonization gender diversity, which has always been there is now resurging.^80^, ^81^ The Midwifery Council of Aotearoa New Zealand's decision to move from ‘woman‐centred’ language to center whānau (family) is an example of a decolonizing approach based on Te Tiriti o Waitangi (the Treaty of Waitangi). Through this process, it was proposed that, for Māori culture, whānau (family) was a more appropriate word than woman to be at the center of care, consistent with mātauranga paradigms of holism.^3^, ^5^, ^82^

An essentialist and colonialist reading of gender rejects the existence of gender‐diverse people before the 20th century, when gender identity was first cataloged,^83^ and later expanded and deconstructed by queer theorists.^84^ The lack of trans visibility in historical texts does not, however, prove that gender non‐conforming people are a recent invention. Heyam^83^ offers a set of global histories to demonstrate that there have always been people who disrupt norms, and there have always been societies in which such norms have never existed. Heyam further argues that it is transphobic to suggest that “trans people are new” as it implies that they are, therefore, not legitimate.^83^ Consequently, puritanical linguistic conservatism and essentialism—resisting change coupled with a privileging of biology in collective identities sited in the word “woman”—harms those who have always existed but have only recently asserted language (e.g., trans and non‐binary) that makes them visible to society.

Essentialist feminism broadly posits that gender is immutable and that it “reflects a natural difference between men and women that is as much psychological, even linguistic, as it is biological”^85^ (p766). This is in contrast to the understanding that gender *varies across time, place, and cultures and is* socially constructed by those with the most privilege to be hierarchical in nature.^86^ Essentialist feminism also differs from radical feminism, which is trans‐inclusive and does not favor biological and sex‐based essentialist theories as set forth in the work of many radical feminist opinion leaders including Monique Wittig, Andrea Dworkin, and Catharine MacKinnon.^87^ Sex and gender essentialism have historically been integral to both first‐wave feminism, and the second‐wave feminism of the 1960s.^88^ Yet, feminist understandings have since evolved. Consequently, some essentialist feminists and others conservative to feminist progress may express concern that queer theory,^84^, ^89^ and intersectional approaches^51^, ^52^ are now imposing themselves in a world where feminist struggle is seemingly unproblematized within a sex/gender binary. However, it is perhaps more accurate to say that it is conservative, white, patriarchal cisheteronormativity that has imposed itself in this colonial context.^2^ There are also echoes of cultural imperialism, hypocrisy, and ideological absolutism apparent in the assumption that conservatives can impose their own reductive ideologies upon the world with regard to reproduction and reproductive health, rather than embracing and adapting to the reality of diversity. Indeed, the idea that it is the so‐called Western, Educated, Industrialized, Rich, and Democratic (WEIRD),^90^ populations who must be responsible for “educating” the global majority on what is “natural” or “normal” in human reproduction is analogous to historical notions grounded in whiteness that purport the superiority of European cis heterosexual masculinity and monogamy.^91^ Equally, such ideas draw back to the European ordering of an assortment of cultures into a single, global narrative and the marshaling of a sustained image of the “natural” patriarchal family.^92^ If we are to decolonize midwifery and reproductive services, we must first deconstruct that which has been imposed. The essentialist idea of gender binary as a universal phenomenon should not only be understood as biologically determinist, but as originating from European patriarchy, which uses violent masculinity as an instrument to marginalize and suppress. Thus, contemporary feminist essentialist ideologies are now recognized to be simply a product of conditioning under patriarchy.^85^

One explanation for resistance to inclusivity in perinatal services is that it poses a threat to otherwise secure colonial and patriarchal power structures. Indeed, another gender essentialist argument is that allowing gender‐diverse people access to women/female‐only spaces exposes cisgender women to an increased risk of violence. Yet, the implied focus here on gender‐diverse people is misguided, as concerns more accurately relate to potential violence perpetrated by cisgender men. Gender‐diverse people continue to experience disproportionate rates of violence when compared with their cisgender peers.^28^, ^93^ The misunderstanding is furthermore that masculine violence is reduced to male biology, rather than being reproduced through colonial and patriarchal gender binaries.^58^ Hence, the cause of “male” violence should be shifted from the biology of “maleness” to the structural environments in which colonial and patriarchal masculinities are reinforced. We may then understand colonial masculinity as a recent formation, and its violence therefore *not* as natural, necessary, or permanent, any more than is colonization itself.^91^ In this way, we may more usefully deconstruct these masculinities in pursuit of reduced societal violence overall. When we understand that violence is both patriarchy‐enhancing and patriarchy‐facilitated,^94^ and that violence is both cause and consequence of gender in coloniality,^73^ we also understand once again that it is the patriarchal gender binaries we need to deconstruct, rather than battle within.

Knowing that “all politics are reproductive politics”, we can see how the colonial configuration of the gender binary is used to produce a society that fits hegemonic understandings of the nation‐state, the nuclear family, and the global population.^95^ As most perinatal care is provided by midwives,^96^ they are ideally placed to cultivate change and progress in decolonizing midwifery and reproductive services. In helping to dismantle the gender binary, patriarchy is destabilized, increasing the reach of reproductive justice for all.

## 7 MIDWIFERY, ESSENTIALISM, AND REPRODUCTIVE JUSTICE

Reproductive justice is a critical feminist and rights‐based framework that acknowledges interconnected and intersectional systemic barriers to care.^8^ Its three central principles include the right to have a child; the right to not have a child; and the right to raise children in safe and healthy environments.^95^ Intersectional approaches in addition to decolonization enable reproductive justice to flourish.^97^, ^98^ The harm caused by the linguistic exclusion of people who do not identify as cisgender women from essential reproductive healthcare is a violation of reproductive justice and thus perpetuates reproductive injustice. Whilst purporting to safeguard the birthing space for cisgender women, we argue that linguistic exclusion actually oppresses and confines them within the patriarchal systems rooted in the gender binary.

Midwives, along with other registered health professionals, have an ethical duty to uphold rights, and are responsible for providing all pregnant people with safe individualized care, respecting bodily autonomy, and working as evidence‐based practitioners.^99^, ^100^, ^101^ Professional responsibilities encompass (1) provision of compassionate, holistic, and culturally safe perinatal care focused on individual needs; (2) upholding rights to self‐determination and bodily autonomy for all; and (3) having expertise and competence to care for a diverse population achieved by continuous education and the application of research and evidence.^99^, ^100^, ^101^ As the World Health Organization asserts,^102^ this includes gender‐diverse pregnant people.

Intrinsic to all healthcare practice is the demonstration of ethical competence through minimizing harm^103^—the bioethical principle of non‐maleficence. In healthcare, harm includes presenting barriers to accessing care, and perpetuating stigmas and discrimination. Such misconduct is common in perinatal services for gender‐diverse communities,^2^ though the cultivation of ethical thinking and adherence to the principles of confidentiality, autonomy, advocacy, respect, and disclosure in relation to gender‐diverse communities have been widely recommended.^104^ Where such failings result in a loss of public trust, further risk ensues as the public is dissuaded from entrusting midwives. Equally, the bioethical principle of justice in this context affirms that transgender people are entitled to equal healthcare access.^105^ In this sense, no one birthing group should be centered in favor of another, and the use of inclusive language is an ethical obligation.

The bioethical principles of autonomy, beneficence, non‐maleficence, and justice must be considered throughout perinatal services.^106^ Yet, while bioethics may be understood as a philosophy invested in the ethical inquiry and practice of the health sciences, and given what we have outlined above, the bioethical imagination is observably lacking in reaching beyond hetero and cisnormativity.^107^ Thus, it may be that bioethics as a framework is no longer wholly useful, given the patriarchal and colonial assumptions behind notions of “autonomy”, for example, and that midwifery practice and maternity services need to look at more politicized ethical praxis, such as those offered by care ethics.^9^, ^10^, ^11^ In addition, queer bioethics,^12^ which enables the appraisal of established bioethical concerns from a queer perspective, maybe a more useful lens with which to actualize “good care” in reproductive health going forward.

A call for a stable and consistent meaning of the word “woman” within midwifery and perinatal services, always understood as rooted in a reproductive biology, is predicated on the notion that language cannot evolve to encompass more categories of sex/gender, and that to do so is inherently dangerous to cis women. Yet, this does disservice to the dynamism that midwifery has been able to display to meet the evolving needs of increasingly diverse childbearing communities. Furthermore, it reduces its ability to privilege reproductive justice over conservatism. Sex/gender should not be the only signifier for potential health inequalities: midwives already adopt a universal and sensitive approach to collecting information from all service users, whilst challenging implicit biases. Categories can—and do—expand, and sex/gender should not be immune to this. Midwifery has a proud history of being inclusive and adaptive to change. Conversely, a failure to evolve in this area may be seen as a regressive step—reinforcing the patriarchal structures in which binaries serve to reinforce dominant norms of who matters, how people must behave and what they must do in relation to childbirth.

Cisgender women are also an oppressed group. Therefore, it is understandable that they, and the midwives believing themselves to be “with woman”, would want to continue to exclusively center cis women in childbearing, particularly in a world where they are de‐centered in seemingly every other domain. Some aspects of the sexed language argument are rooted in utilitarianism (i.e., the greatest good for the greatest number) because most of the birthing people are cisgender women. Certainly, the centering of “woman” and related etymology (e.g., maternity/maternal) may appear logical in this context. Yet, as we have argued, maintaining exclusively “woman‐centred” rather than “person centred” reproductive language actually further oppresses cis women by maintaining the gender binaries rooted in patriarchal and colonial oppression. Utilitarian approaches in healthcare are also known to widen inequalities rather than address them.^108^, ^109^ Ultimately, utilitarianism fails to consider justice, and so even if the centering of “women” in childbirth did produce great benefits, injustice would remain.

Whilst people can always state their preferences for language in how they wish to be addressed on an individual basis, suggesting additive or expansive language (e.g., women and people who birth) on a systemwide scale may not enable true inclusivity as it further entangles “women/woman” and “pregnancy/birth” as necessarily connected terms, and problematically upholds the feminization of pregnancy. Furthermore, this approach always names ‘women’ and then only adds “others.” Consequently, it reproduces women as the default, and *others* the rest of people who might be pregnant into one “added” category. There is no justice in naming women explicitly whilst not also naming other gender identities explicitly. Centering “pregnant people” as the norm in reproductive/perinatal services, guidelines, and practice does not erase cis women. It includes them within a broader category to ensure that no single gender is centered on reproduction to avoid further upholding patriarchal ideals. With the need for wholly inclusive language in perinatal and midwifery services firmly established, we offer alternatives to exclusive language as presented in Table 1.

**TABLE 1:** A guide to gender‐inclusive language in perinatal and midwifery services.

Level/Terminology		Exclusive		Inclusive	Examples of inclusive terminology
Individual		Assuming the gender of the individual receiving care and/or support person(s).		Ask the individual their name, pronouns, and words they want to use for body parts and parenting roles (e.g., dad/mum/hijra). Use this terminology. Mirror the language they use	“Hello. My name is [X] and my pronouns are [Y]. What's your name and what are your pronouns?” “How do you want to be called as a parent?” “How do you intend to feed your baby?” *[mirror language used in their answer]* “Who have you brought with you today?” *[mirror language used in their answer]*
				Ask for the name, pronouns, role, and relationship of any support person(s) present. Use this terminology. Mirror the language they use	“Who have you brought with you today?” “Hello. My name is [X] and my pronouns are [Y]. What's your name and what are your pronouns?”
Population	People	Women/mothers/mums		Pregnant people	Pregnant people should be supported to access antenatal care
				Pregnant population	The pregnant population should be supported to access antenatal care
				Service users	Service users should be supported to access antenatal care
				Expectant parents	Expectant parents should be supported to access antenatal care
				Gestational parents	Gestational parents should be supported to access antenatal care
				Anyone who is pregnant	Anyone who is pregnant should be supported to access antenatal care
				*Restructure for direct address*	In pregnancy, you should be supported to access antenatal care
		Men/fathers/dads		Co‐parents	Co‐parents are welcome at antenatal classes
				Non‐gestational parents	Non‐gestational parents are welcome at antenatal classes
				A member of your support team	A member of your support team is welcome at antenatal classes
				*Restructure for direct address*	As a support person, you are welcome to antenatal classes
		Maternal	*[adjective]*	Parental *[if another parent is not present]*	Check parental blood pressure
				Pregnant/birthing/postnatal *[choose as appropriate]*	Postnatal depression affects different people in different way
				Gestational parents'	Gestational parents' outcomes are a priority
		Maternal	Maternal consent	Informed consent	Informed consent given
				[Name's] consent	[X's] consent given
				Parental consent *[if another parent is not present]*	Parental consent given
			Maternal notes	Handheld notes/records	The telephone number is on the front of the handheld notes
				Care record	The telephone number is on the front of the care notes
				Antenatal/intrapartum / postnatal notes *[choose as appropriate]*	The telephone number is on the front of the antenatal/intrapartum / postnatal notes *[choose as appropriate]*
		Woman‐centered		Person‐centered	We provide person‐centered care
				Individualized	We provide individualized care

	Feeding	Breastfeeding		Breast‐ and chestfeeding	A calm environment facilitates breast‐ and chestfeeding
				Breast/chestfeeding	A calm environment facilitates breast/chestfeeding
				Body feeding	A calm environment facilitates body feeding
				Human milk feeding	A calm environment facilitates human milk feeding
				Human milk provision	A calm environment facilitates human milk provision
				Lactation	A calm environment facilitates lactation
		Breastmilk		Breast‐ and chest milk	Breast‐ and chest milk houses its own microbiome
				Breast/chest milk	Breast/chest milk houses its own microbiome
				Human milk	Human milk houses its own microbiome
Milk from the feeding parent/co‐parent *[choose as appropriate]*				Milk from the feeding parent/co‐parent *[choose as appropriate]* houses its own microbiome
System and service	Places	Maternity		Perinatal services	Perinatal services strive to provide positive experiences
				Perinatal care services	Perinatal care services strive to provide positive experiences
				Reproductive health services	Reproductive health services strive to provide positive experiences
				Gestational health services	Gestational health services strive to provide positive experiences
				Pregnancy/birth/postnatal/infant feeding services *[choose as appropriate]*	Infant feeding services strive to provide positive experiences
		Women's center/hospital		Perinatal care services/center/hospital	Your local perinatal care hospital is open 7 days a week
				Reproductive health services/center/hospital	Your local reproductive health services are open 7 days a week
				Gestational health services/center/hospital	Your local gestational health services are open 7 days a week
	Professional roles and titles	Matron		Executive Perinatal Lead	The Senior Perinatal Lead is in charge of the ward
		Sister		Senior Perinatal Lead	The Executive Perinatal Lead has written this policy
		Midwife		Lead perinatal practitioner	At the first appointment, your lead perinatal practitioner will ask to take blood to check your blood group
		Gynecologist (*Gynae* translates to *woman* in Greek)		Reproductive Health Specialist	We can refer you to see the Reproductive Health Specialist
	Parenting groups	Mum and baby classes		Antenatal/birth preparation/infant feeding classes *[choose as appropriate]*	You can meet other expectant parents at our birth preparation classes
				Parenting groups	You can meet other parents at our parenting group
				You and your baby class	You can meet other parents at the “You and your baby class”

*Note*: Terminology to avoid: (1) Asterisk with a disclaimer, e.g., “This policy acknowledges that not all people who use our service are women, and the word “women” will be used throughout to refer to all who give birth.” (2) Adjective “other”, e.g., “This policy is for women and other pregnant people”. (3) Language that centers women at population and system levels (e.g., “This policy is for women and pregnant people”).

## 8 DISCUSSION AND CONCLUDING POINTS

In this paper, we have argued that the protection of the marginalized profession of midwifery, understood as “women being with‐woman”, risks becoming deeply wound up with an essentialist understanding of sex that is ultimately white, heteronormative, patriarchal, and colonial. ‘Sex‐based’ excluding language only perpetuates this essentialist thinking. Moreover, to insist on exclusive sexed language in childbearing, is to uphold a racial patriarchy that does not align with the values of midwifery ethics and/or reproductive justice. We demonstrate how the adoption of an inclusive approach would benefit all who birth, the majority of whom are cisgender women.

Much like the fallacy we have previously described in relation to cisgender women being protected by spaces which exclude trans and non‐binary people, we have shown how essentialism in midwifery similarly stems from a misguided protection of the marginalized profession of midwifery, which is also ultimately tied to a colonialist understanding of gender. To understand sexed language as fundamentally tied to the protection of the profession of midwifery is a historical and feminist misconception which does ultimately the opposite: it undoes any claim that midwifery might have on reproductive justice and feminist ethics. Sex/gender are not inevitably binary, nor can they be considered independent of each other; rather, they are structurally reproduced in social systems and organizations. Upholding sexed language in midwifery should not, therefore, be understood as “effective” but offers an opportunity to explore the harm that it reproduces in a society committed to patriarchal and colonial values. If midwifery aims to work by the principles of reproductive justice, it has an ethical imperative to use wholly inclusive language. Moreover, this inclusive approach would undoubtedly be of value to other areas of practice besides language.

Given the imperative for whole gender‐inclusive language in perinatal and midwifery services, we offer the first specific guidance on this, building on broader guidance published elsewhere.^3^, ^110^ If midwifery is indeed a feminist profession, it, therefore, follows that it should reject any re‐affirmation of a European patriarchal sex binary rooted in colonialism, and fight for reproductive justice to the benefit of all who birth, the majority of whom are cisgender women. This starts with language.

## CONFLICT OF INTEREST STATEMENT

The authors have no conflicts of interest to declare.

## Data Availability

Data sharing not applicable to this article as no datasets were generated or analysed during the current study.
